# Complement regulation in the eye: implications for age-related macular degeneration

**DOI:** 10.1172/JCI178296

**Published:** 2024-05-01

**Authors:** Georgia A. Wilke, Rajendra S. Apte

**Affiliations:** 1 John F. Hardesty, MD, Department of Ophthalmology and Visual Sciences,; 2 Department of Medicine, and; 3 Department of Developmental Biology, Washington University School of Medicine, St. Louis, Missouri, USA.

## Abstract

Careful regulation of the complement system is critical for enabling complement proteins to titrate immune defense while also preventing collateral tissue damage from poorly controlled inflammation. In the eye, this balance between complement activity and inhibition is crucial, as a low level of basal complement activity is necessary to support ocular immune privilege, a prerequisite for maintaining vision. Dysregulated complement activation contributes to parainflammation, a low level of inflammation triggered by cellular damage that functions to reestablish homeostasis, or outright inflammation that disrupts the visual axis. Complement dysregulation has been implicated in many ocular diseases, including glaucoma, diabetic retinopathy, and age-related macular degeneration (AMD). In the last two decades, complement activity has been the focus of intense investigation in AMD pathogenesis, leading to the development of novel therapeutics for the treatment of atrophic AMD. This Review outlines recent advances and challenges, highlighting therapeutic approaches that have advanced to clinical trials, as well as providing a general overview of the complement system in the posterior segment of the eye and selected ocular diseases.

## Introduction

Investigation into ocular disease must account for the eye’s unique relationship with the systemic immune system. The eye is an immune-privileged organ, meaning it can tolerate novel antigens without launching an inflammatory response (1, 2). This privilege is maintained by multiple mechanisms, including physical obstacles such as the blood-retina barrier, and the presence of immunosuppressive factors within the intraocular milieu (3). Complement signaling in the context of antigen presentation within the eye also leads to suppression of antigen-specific immune responses (4). Immune privilege is vital to eye function; it protects the visual axis from damage due to unregulated innate and adaptive immune activity so that light can reach the neurosensory retina unimpeded.

While the eye resists exuberant inflammation, it is susceptible to parainflammation, a state of low-grade inflammation in response to cellular stress that helps restore and maintain tissue functionality (5–7). Both immune privilege and parainflammation require the sophisticated regulation of the complement cascade, a central part of innate immunity that can cause severe intraocular inflammation if left unchecked (8). The neurosensory retina is particularly susceptible to damage from inflammation due to its complex structure and limited regenerative potential. It is therefore not surprising that complement dysregulation specifically within the retina contributes to the pathogenesis of many ocular diseases (9, 10); investigation into these diseases has led to an enhanced understanding of the role of complement in the eye. In this Review, we focus on the contribution of complement to the pathophysiology of age-related macular degeneration (AMD), highlighting the clinical development of complement-targeting therapeutics for dry AMD.

## Overview of the complement system

**Figure 1:**
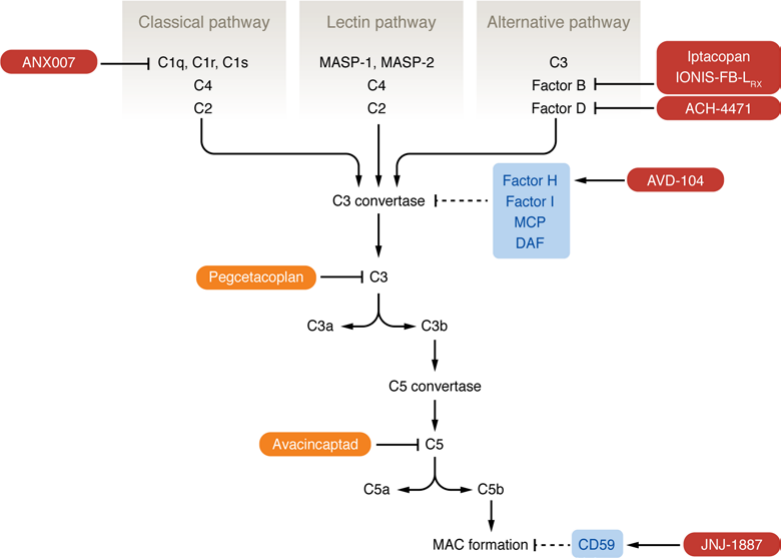
Pathways of the complement system. The complement system is composed of three pathways (classical, lectin, and alternative) that converge on the formation of a C3 convertase complex that is unique to each pathway. The classical pathway begins with binding of the C1 complex (composed of C1q, C1r, and C1s) to an antigen-antibody complex of pathogen surface directly; this leads to cleavage of C4 and then C2 to form the C3 convertase of the classical pathway. The lectin pathway is similar in that it begins with MBL recognizing mannose residues on a pathogen surface; this activates the MBL-associated serine proteases MASP-1 and MASP-2, which cleave C4 and C2. The alternative pathway is initiated by spontaneous hydrolysis of C3, which binds FB, leading to cleavage of FB by FD; this complex is stabilized by properdin. C3 convertase activity leads to the formation of the C5 convertase and eventually the MAC, triggering membrane destabilization of foreign material. Host complement inhibitors (light blue: FH, FI, MCP, DAF, CD59) target C3 convertase and MAC formation. FDA-approved complement inhibitors for GA (orange) are pegcetacoplan, which targets C3, and avacincaptad, which targets C5. Investigational therapies for GA (dark red) target components of the classical and alternative pathways.

The complement system is a network of proteins that function as part of innate immune surveillance (11–13); for a thorough review of the systemic biology of complement and how complement dysregulation contributes to pathology, see Mastellos et al. (14). The complement system encompasses the classical, lectin, and alternative pathways — which are triggered by distinct mechanisms — and converges on the cleavage of C3, the central molecule of the complement network, leading to C5 activation and initiation of the terminal lytic pathway (Figure 1). The C3 convertase of each pathway cleaves C3 into active fragments C3a and C3b, which act as anaphylatoxins and opsonins, respectively. C3 convertase activity directly leads to creation of the C5 convertase, which cleaves C5, creating C5a and C5b. C5a is an anaphylatoxin, while C5b, in a complex with C6 and C7, binds to membrane surfaces and initiates the assembly of the membrane attack complex (MAC), causing membrane destabilization.

The three complement pathways arrive at the creation of the C3 convertase differently (11–13). The classical and lectin pathways are similar in that pattern recognition molecules bind to a surface and then trigger complement activation. In contrast, the alternative pathway is continuously active at low levels. In a process called “tick-over,” C3 spontaneously hydrolyzes into C3(H_2_O), which binds factor B (FB), leading to cleavage of FB by factor D (FD). The resulting complex is stabilized by binding to properdin, a soluble positive regulator of the complement system. This series of reactions creates the C3 and C5 convertases of the alternative pathway.

Host cells inhibit complement activation through the expression of specific inhibitory proteins (11–13). These proteins include factor H (FH), a soluble inhibitor of the alternative pathway C3 convertase; and membrane cofactor protein (MCP) and decay-accelerating factor (DAF), two membrane-bound inhibitors that also prevent C3 convertase formation.

## Overview of the posterior segment of the eye

**Figure 2:**
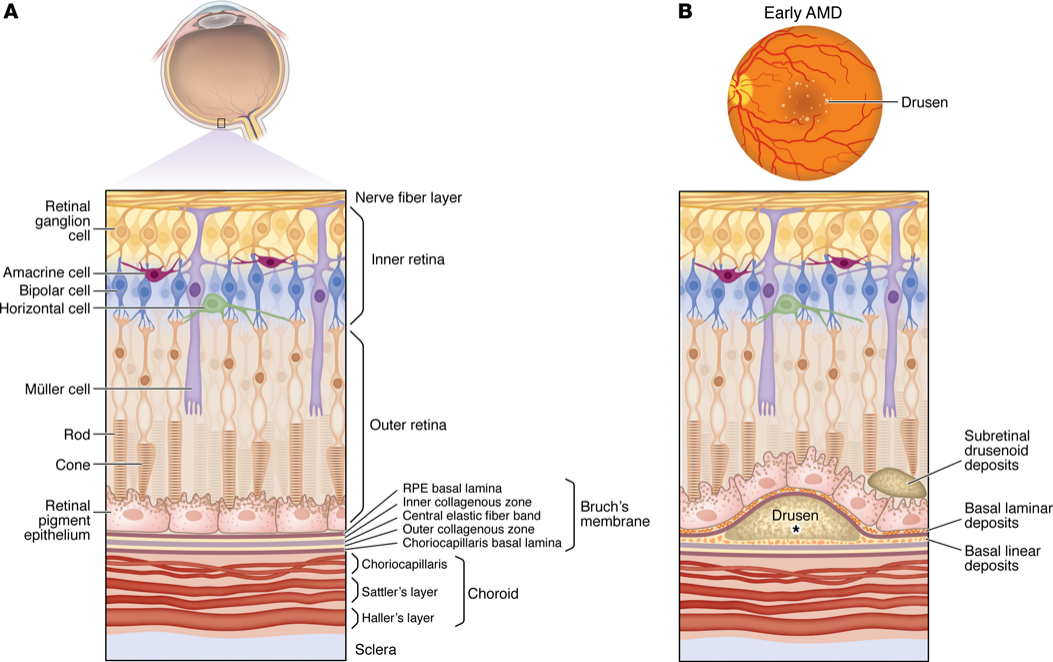
The retina consists of specialized cell types organized into layers. (**A**) The outer retina consists of photoreceptors, while the inner retina contains bipolar, amacrine, horizontal, Müller, and retinal ganglion cells. Bipolar cells synapse with photoreceptors and transmit their signal to ganglion cells. Horizontal and amacrine cells regulate photoreceptor and bipolar cells, respectively. Müller cells are the glial/support cells of the retina. The retina is supported by the retinal pigment epithelium (RPE). The basal lamina of the RPE forms part of Bruch’s membrane (BM), a multilayered ECM. (**B**) Pathological changes in early AMD occur in BM and the RPE. Basal laminar deposits appear between the RPE and the RPE basal lamina; basal linear deposits form in the inner collagenous zone of BM. Drusen are deposits beneath the RPE basal lamina; they contain cellular debris, including lipids, proteins, and complement components, such as C3, C5, and MAC (denoted by the asterisk) (74). Subretinal drusenoid deposits form anterior to the RPE and are associated with an accelerated neurodegenerative phenotype in AMD.

The retina is a multilayered structure consisting of specialized cell types that enable the conversion of a light stimulus to an electrical impulse. The outer/posterior layer of the retina consists of photoreceptors. The inner/anterior layer consists of accessory and bipolar cells that transmit the photoreceptor signal to retinal ganglion cells (Figure 2A) (15, 16). Microglia also reside within the retina and play key immune surveillance roles (17, 18).

Photoreceptors are supported by a monolayer of specialized cells called the retinal pigment epithelium (RPE). The RPE has many functions, including maintenance of retinal adhesion, vitamin A metabolism, and recycling the byproducts of phototransduction (19). Posterior to the RPE is Bruch’s membrane (BM), a thick extracellular matrix (ECM) (Figure 2) (19). The RPE and BM form the *outer* blood-retinal barrier, preventing passage of immune cells and large molecules from the choroid into the neurosensory retina. The *inner* blood-retinal barrier is formed by the endothelium of the inner retinal vasculature.

The choroid, located between the sclera and retina, provides blood supply to the RPE and outer retina. The choroid is outside the blood-retina barrier and is part of the systemic circulation. Part of the choroid is the choriocapillaris, a layer of small-diameter fenestrated vessels that lies just posterior to BM (Figure 2A) (20). The fenestrations of the choriocapillaris facilitate delivery of nutrients to and removal of waste products from the RPE and photoreceptor cells.

## Complement activity in the healthy eye

Early studies of intraocular complement activity revolved around immunohistochemical localization of complement components within ocular structures and quantification of complement activity within ocular fluids by use of in vitro assays. Animal studies have demonstrated the presence of C3 cleavage fragments within homogenized intraocular tissue and MAC deposition in the choroid, indicating low-level flux through the terminal complement pathway (8). This activity is tightly regulated by inhibitory proteins such as MCP and DAF, as chemical- or immune-mediated inhibition of the equivalent proteins in rat eyes led to severe intraocular inflammation (8). The presence of inhibitory proteins was demonstrated in human eyes by immunohistochemical studies showing DAF in the retinal nerve fiber layer and MCP in the basolateral surface of the RPE (21, 22). Additionally, the MAC-inhibiting protein CD59 is localized to the cornea in addition to the retina and choroid in human eyes (23). FH was found mostly in the choriocapillaris, while cofactor FI localizes to the inner retina (24). Human aqueous and vitreous fluid inhibit both the classical and alternative complement pathways in vitro (25). Taken together, these studies point to a basal level of complement activity within the eye that is kept in balance by the presence of complement-inhibitory proteins.

Constitutive gene expression of components of all complement pathways have been detected in isolated murine retina, RPE, and choroid (26). In vitro experiments with murine RPE cells demonstrate upregulation of components of the alternative and classical pathways in response to the inflammatory cytokines IFN-γ and TNF-α. Interestingly, TNF-α leads to downregulation of FH in RPE cells (26). Studies using cultured human RPE cells demonstrated that protein expression of MCP, DAF, and CD59 increases in response to TNF-α or IL-1β (27). These studies suggest dynamic regulation of expression of complement-inhibitory proteins in the posterior segment of the eye in response to inflammatory signaling.

Complement activity in the eye goes beyond immune surveillance; it also plays a role in the creation of tolerance to antigens originating in the eye, thereby supporting the immune-privileged state of the eye. Antigen introduced into the anterior chamber of the eye triggers development of antigen-specific regulatory T cells and suppression of the delayed-type hypersensitivity (DTH) response, a type of T cell–mediated immunity. This immune-suppression phenomenon is called “anterior chamber–associated immune deviation” (ACAID) (28). Animal studies have shown that the complement system is required for ACAID; rats depleted of complement by administration of cobra venom factor as well as C3-deficient mice were unable to suppress DTH to antigen injected into the eye (4). ACAID was found to be dependent on iC3b — a cleavage fragment of C3b — binding to its receptor, CR3, on antigen-presenting cells (APCs). This binding leads to secretion of TGF-β2 and IL-10, two cytokines that suppress DTH, by APCs. Therefore, complement not only acts as a first line of defense against pathogens in the eye due to its chronic low level of activity, but it also protects the eye from the destructive effects of T cell–mediated inflammation.

## Complement activity in ocular disease

Fine control of intraocular complement activity is critical for avoiding unnecessary inflammation that would degrade the visual axis. Complement dysregulation has been implicated in the pathogenesis of many ocular diseases, which has been extensively described in multiple reviews (10, 29–31). We highlight several diseases listed in Table 1 and then focus on a comprehensive discussion of complement activity in AMD, given the recent translational developments and approval of complement-based therapies.

### AMD.

AMD is a progressive neurodegeneration of the retina that causes central vision loss. Late-stage AMD is functionally debilitating and is associated with impaired ability to perform activities of daily living (32). It is a disease of the elderly; therefore, the global burden of AMD is projected to rise as the aging population increases, with 300 million people projected to be diagnosed with AMD by 2040 (33). Complement activity has been a major focus in the investigation into AMD pathogenesis, leading to better understanding of the role of complement in the aging retina and the development of novel therapeutics (14, 29, 34, 35).

Pathological changes in AMD involve the photoreceptors, RPE, BM, and choriocapillaris. The earliest lesions are detectable by histology or electron microscopy; these are abnormal deposits within the RPE-BM complex called basal laminar deposits (BlamD) and basal linear deposits (BlinD) (Figure 2B). BlamD consist of lipid-rich material and collagen fibers and are found between the plasma membrane and basal lamina of the RPE cells; they are associated with dysmorphic overlying RPE (36). BlinD are phospholipid vesicles with electron-dense granules within the inner collagenous zone of BM (37), i.e., posterior to the RPE basal lamina (38, 39). Anatomical studies have shown that BlamD and BlinD are found more frequently in eyes with AMD compared with age-matched controls (36, 40, 41).

The first clinically evident lesions in AMD are “drusen,” extracellular deposits that appear posterior to the RPE basal lamina (Figure 2B). Drusen are related to BlinD due to their shared location (36) and are visible on fundus examination as round, yellow lesions in the macula. Drusen are aggregates of proteins, lipids, and cellular debris; major components include albumin, apolipoprotein E, complement factors, and immunoglobulin (42–44). In addition to sub-RPE drusen, subretinal drusenoid deposits are also seen in AMD patients and are associated with an accelerated neurodegenerative phenotype (Figure 2B) (45).

AMD progression is categorized into stages that are based on the size of drusen (46). The Age-Related Eye Disease Study (AREDS) found that patients with early AMD (many small drusen or few intermediate drusen) had a low risk (1.3%) of progressing to late AMD over a 5-year period, while patients with many intermediate drusen or a single large druse had an 18% chance of converting to late AMD over the same period (47). Late AMD is divided into two forms: neovascular (wet or exudative) AMD (nvAMD) and advanced dry (atrophic) AMD (48).

### Neovascular AMD.

**Figure 3:**
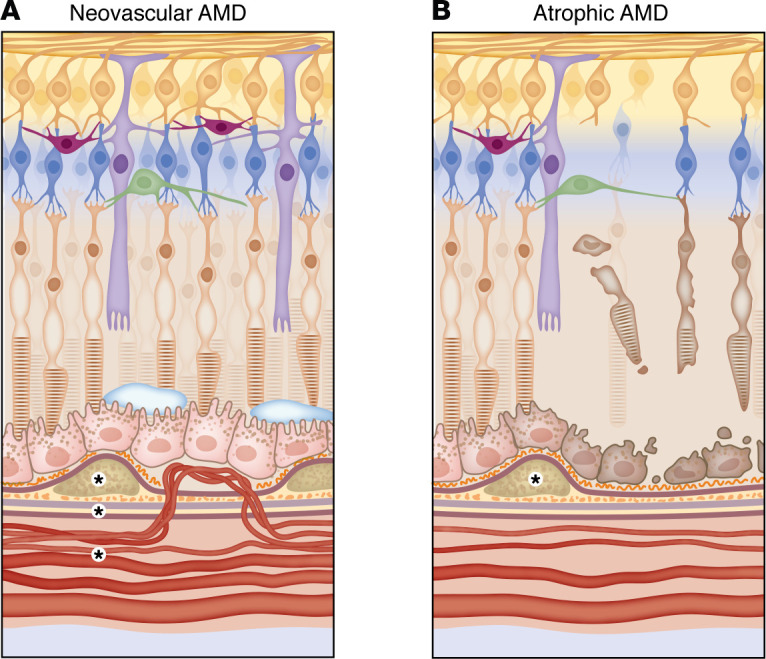
The retina in AMD. (**A**) In neovascular AMD, it is hypothesized that choriocapillaris atrophy leads to ischemia of the RPE, which triggers VEGF secretion and the growth of abnormal choroidal blood vessels. These vessels breach BM and grow in the sub-RPE or subretinal space, causing accumulation of subretinal and intraretinal fluid. (**B**) In atrophic AMD, it is thought that some primary insult leads to RPE degeneration, which causes choriocapillaris atrophy due to the role of the RPE in supporting choriocapillaris function. As the RPE degenerates, the overlying photoreceptors die. In both types of AMD, there is choriocapillaris atrophy and RPE degeneration, though the sequence of events in each disease may be different. In terms of complement activity in AMD, increased concentrations of C3, C3a, Bb, FB, and FD have been detected within BM and choriocapillaris of human donor eyes with AMD (denoted by asterisks) (100). Cadaver studies have found MAC deposition in the RPE and choriocapillaris of patients with the Y402H polymorphism in *CFH* regardless of whether AMD changes are present (88, 89). The Y402H polymorphism is believed to contribute to AMD pathogenesis primarily through its effect on FHL-1, as FHL-1 is the major complement regulator of BM (83, 84).

The defining feature of nvAMD is the development of choroidal neovascularization (CNV) (Figure 3A). The trigger for CNV is unknown; it is thought that the pathologic changes in early dry AMD create a proangiogenic environment (49–51). According to one hypothesis, the atrophic changes in the choriocapillaris (52) and decreased choroidal blood flow seen in AMD patients (53) leads to hypoxic RPE, which then secretes VEGF (54), leading to CNV (55). In support of this, a study with donor eyes found areas of choriocapillaris degeneration adjacent to active CNV, suggesting that the RPE overlying these regions was hypoxic and creating the VEGF stimulus that led to CNV growth (55).

The abnormal vessels of CNV may breach BM and grow into the sub-RPE space or they may grow in the space between the RPE and neurosensory retina (56). The vessels leak, leading to subretinal and intraretinal fluid that distorts vision (Figure 3A). Left untreated, the vessels fibrose, creating a disciform scar, leading to central vision loss (56).

Treatment of nvAMD revolves around VEGF inhibition. All current therapies for nvAMD are delivered via intravitreal injection and usually require administration every 1–3 months over many years. Multiple anti-VEGF therapies are currently in use and these have revolutionized the treatment of nvAMD; the vast majority of patients can now maintain their vision within 3 lines of their presenting visual acuity (57).

### Atrophic AMD.

Atrophic AMD and its advanced form geographic atrophy (GA) are clinically apparent as an area of RPE loss through which the large choroidal blood vessels are visible (Figure 3B). Histologically, there is RPE degeneration with loss of associated photoreceptors and choriocapillaris (55). Atrophic lesions can be unifocal or multifocal, and the area of atrophy typically increases at a rate of 1.5–2 mm^2^ per year, though this can vary substantially depending on the location of lesions and presence of environmental risk factors such as smoking (58, 59). Once atrophy involves the fovea, severe central vision loss occurs.

It is unclear in atrophic AMD where the initial damage occurs. Some cadaver studies suggest that in GA, RPE death occurs first, followed by loss of the outer retina and choriocapillaris (55). Remaining vessels have reduced diameter and decreased number of endothelial fenestrations (55). This change in the choriocapillaris after RPE atrophy likely occurs because the RPE supports endothelial function by secreting VEGF (60, 61) — an endothelial cell survival factor (62) that induces fenestration formation (63) — helping to maintain the highly permeable nature of the choriocapillaris. When the RPE degenerates, the VEGF signal is lost, and the choriocapillaris atrophies. This sequence of events (RPE degeneration leading to choriocapillaris atrophy) is opposite to the proposed pathologic changes that occur in nvAMD (choriocapillaris atrophy leading to ischemic RPE); it is likely that both mechanisms exist and represent different outcomes of complex pathology. Other studies using human donor eyes with early AMD show an inverse relationship between the total area of drusen deposits and choriocapillaris vascular density with the RPE remaining intact (64), i.e., vessel atrophy is associated with a greater drusen burden. This suggests that vessel atrophy underlies both wet and dry AMD.

The nature of the primary insult to the RPE in dry AMD is unknown but is likely multifactorial, involving an interplay of environmental and genetic factors. Oxidative stress has been proposed to be a contributor to RPE damage in AMD (65) and was implicated by the results of the AREDS trials, which showed that in patients with intermediate AMD, antioxidant supplementation reduced the risk of progression to late AMD (47, 66). Aging in general is associated with the accumulation of oxidized lipids and proteins in the retina (67), as well as advanced glycation end products that interfere with RPE and BM function (68). Animal models of photooxidative stress demonstrate activation and migration of resident tissue macrophages to the outer retina, accompanied by C3, FB, and MAC deposition in the outer retina and RPE (69, 70), suggesting that complement activation plays a role in the initial damage and recovery from oxidative stress.

It is hypothesized that the increasing oxidative damage with age leads to parainflammatory activity in the retina to restore homeostasis (6, 67); indeed, animal models of aging have shown increased inflammatory gene and protein expression in the retina and choroid (71, 72). Microglia isolated from the retina of aged mice show increased expression of C3 and FB; there is also increased deposition of C3 and FB in the outer retina of senescent mice (73). This age-related change in immune activity in the retina creates the environment in which the pathologic changes associated with AMD occur; AMD may represent the transition from appropriate parainflammation in response to mild retinal injury to outright immune dysregulation (5, 6)^.^

## Dysregulation of complement in AMD

The first hint that complement activity was involved in AMD pathogenesis was from immunohistochemical studies revealing the presence of C3, C5, and MAC in drusen, which led to the hypothesis that drusen were the consolidated byproducts of local inflammatory activity (74). It was proposed that debris from a primary RPE insult becomes trapped between the RPE basal lamina and the rest of BM, creating a “seed” that stimulates local inflammatory activity (74–76).

These early insights into complement activity in AMD were massively expanded upon by genetic studies published in 2005 that found a link between a variant in *CFH* and AMD (77–80). *CFH* encodes FH, a soluble cofactor for FI-mediated cleavage of C3b that also prevents formation of the alternative pathway C3 convertase (81), and FHL-1, an alternative splicing variant of FH with complement-inhibitory functions (82). FH and FHL-1 are fluid-phase regulators of the alternative pathway and function to regulate complement activation on acellular surfaces (e.g., basement membranes) (82). The genetic studies found that a nonsynonymous point mutation, rs1061170, in *CFH* results in a significant predisposition for AMD. The mutation results in the replacement of a tyrosine residue by a histidine residue at position 402 (Y402H).

The Y402H polymorphism is present in both FH and FHL-1, but it likely predominantly exerts its effect on AMD pathogenesis through FHL-1, as FHL-1 has been demonstrated to be the major complement regulator of BM; its truncated form allows it to passively diffuse through BM, while FH is located in the ECM of the choroid (83, 84). FH and FHL-1 bind to the ECM through the recognition of glycosaminoglycans (GAGs); the Y402H polymorphism occurs at one of the GAG-binding sites (85), and this variant leads to decreased FHL-1 binding to heparan sulfate within BM (86). It is hypothesized that decreased binding of FHL-1 within BM due to the Y402H polymorphism leads to increased complement activation and chronic inflammatory activity that contributes to AMD pathogenesis (87). In support of this, in a study of cadaver eyes, donors homozygous for Y402H exhibited higher levels of MAC in the RPE/choroid compared with patients with a low-risk genotype, regardless of whether the donor eyes had signs of AMD (88). A similar study also found higher levels of MAC deposition in BM and the choriocapillaris, without any associated AMD changes, in donor eyes from patients with the *CFH* risk haplotype (89). These studies suggest that local complement dysregulation far precedes the early pathological changes in AMD.

*CFH* was the first gene to be linked to AMD; in the last two decades, other complement genes have been implicated. In general, variants that enhance complement activation are associated with increased risk, while variants that interfere with complement activity are protective. For example, a specific *C3* polymorphism that is associated with increased AMD risk (90, 91) has been shown to have more efficient complement activation and decreased FH binding (92). A different *C3* allele, which results in decreased C3 inactivation by FH and FI, is also associated with increased AMD risk (93). One *CFB* variant is associated with protection from AMD (94, 95); this variant has demonstrated decreased C3 convertase formation in vitro (96).

The FHR proteins lie downstream of *CFH* on chromosome 1q31 and are mentioned in Table 1 in the context of multifocal choroiditis pathogenesis. Their function is poorly understood, but they are thought to compete with FH binding to C3 and other substrates and thereby interfere with complement inhibition (83, 97, 98). Variants located on 1q31 have been associated with increased levels of circulating FHR proteins in patients with AMD (83, 84), though the effect of this on systemic complement activity is unknown. Interestingly, a haplotype with deletion of *CFHR1* and *CFHR3* is associated with decreased risk of AMD (77, 98, 99).

In general, there is evidence of increased complement activity both systemically and within the eye in AMD patients. Increased concentrations of C3, C3a, Bb, FB, and FD have been detected within BM and choriocapillaris of human donor eyes with AMD (100). Analysis of transcriptome profiles of RPE-choroid isolated from donor eyes of patients with AMD shows upregulation of complement pathway genes (101). Serum levels of complement-breakdown products such as C3d (degradation product of C3b), C3a, Ba, and C5a are elevated in patients with AMD compared with the control group (102), implying increased flux through the alternative complement pathway. In nvAMD specifically, complement may be required for the development of CNV; in a laser injury–induced CNV mouse model, deposition of C3 and MAC in the neovascular complex was observed, and *C3^–/–^* mice did not develop CNV (103).

## Complement targets in atrophic AMD

With the abundance of data pointing toward complement dysregulation as a driver in AMD pathogenesis, current investigations into AMD treatment have focused on targeting complement activity. While we limit our discussion here to therapeutics that have advanced to clinical trials, new therapeutics at all stages of investigation are the focus of several recent reviews (29, 35, 104, 105). Interestingly, complement targets in AMD were the subject of clinical trials over a decade ago. POT-4 (Potentia Pharmaceuticals) — a derivative of compstatin, a peptide inhibitor of C3 (106) — was the first complement inhibitor to be tested in clinical trials for AMD (Table 2). POT-4 was administered to patients with neovascular (107) and dry (108) AMD but did not show benefit in phase II trials. C5 was also investigated as a target in phase I/IIa clinical trials that examined the safety and tolerability of an anti-C5 aptamer in the treatment of nvAMD (109, 110) (Table 2). These programs were not advanced, and study results have not been reported in peer-reviewed publications. See Table 2 for other previously investigated complement therapeutics in AMD. In the last 10 years, there has been considerable interest in the role of complement inhibition in the treatment of atrophic AMD with GA, with several new therapeutic targets emerging in the last five years.

### Targeting C3.

The different complement pathways converge on the creation of the C3 convertase, leading to C5 convertase and MAC formation. Logically then, C3 is an attractive target, as it represents a central hub in the complement cascade. The compstatin family of C3 inhibitors have been at the center of investigation into C3 targeting in AMD for over a decade (111). As mentioned earlier, POT-4, a compstatin derivative, was studied in AMD clinical trials and did not demonstrate significant benefit (107, 108). However, pegcetacoplan (Syfovre, Apellis Pharmaceuticals; Table 3) is a pegylated C3 inhibitor peptide based on a second-generation compstatin derivative (111, 112) that was approved by the US FDA in 2023 for the treatment of GA. Pegcetacoplan binds C3 and prevents its cleavage/activation and also binds C3b, thereby inhibiting the activity of the C3 and C5 convertases of the alternative complement pathway, which contain the C3b subunit (Figure 1). The phase III OAKS and DERBY trials evaluated the efficacy of pegcetacoplan given intravitreally every month or every other month in preventing progression of GA (113). At 24 months, in the OAKS trial, patients receiving pegcetacoplan monthly or every other month had 22% and 18% less growth of GA lesions, respectively, compared with patients in the sham treatment group. The reduction in GA growth rate for the pegcetacoplan-treated groups in the OAKS trial reached statistical significance by 12 months, while in the DERBY trial, significance was not reached until the 24-month time point for analysis of outcomes. The GALE extension study investigated the efficacy and safety of pegcetacoplan over 36 months of continuous treatment; these data were recently presented, and pegcetacoplan continued to show effectiveness in reducing GA growth rate, with the treatment arm demonstrating reduced GA lesion growth of 35% and 24% (monthly and every other month, respectively) compared with the sham arm (114).

### Targeting C5.

The C5 convertase initiates MAC formation, the final effector complex of complement. C5 inhibition in GA was initially explored with the phase II COMPLETE study, which investigated the effect of intravenous administration of eculizumab (Figure 1 and Table 2), an anti-C5 antibody, on GA progression (115). The study found no significant decrease in GA growth rate after 6 months in patients receiving eculizumab (115).

C5 was considered a viable target again with avacincaptad pegol (IZERVAY, IVERIC bio; Table 3), a pegylated RNA aptamer that binds and prevents C5 cleavage/activation (Figure 1). GATHER1 was a phase II/III trial that evaluated the effect of monthly avacincaptad administration via intravitreal injection compared with sham in terms of GA lesion growth; the study found a 28.1% and 30.0% reduction in mean GA growth for patients receiving 2 mg and 4 mg of avacincaptad, respectively, over 18 months (116). GATHER2 was a phase III trial in which patients received either sham or avacincaptad 2 mg monthly for 1 year; after 1 year, the participants in the avacincaptad group were randomized to either continue receiving avacincaptad every month or switch to every other month (117). The recently published 12-month results of the study also demonstrated a significant (14%) decrease in GA lesion growth in the avacincaptad compared with the sham treatment group (117). Avacincaptad recently joined pegcetacoplan in gaining approval by the FDA for treatment of GA secondary to dry AMD (118).

### Safety considerations with targeting C3 and C5.

A common concern with complement therapeutics is the potential risk of infection with systemic or localized complement inhibition. This issue has been extensively discussed in other reviews (35, 119), and fortunately it appears that intraocular infection is rare with intravitreal administration of these drugs, as the trials investigating pegcetacoplan and avacincaptad reported that overall safety profiles were favorable. However, after FDA approval of pegcetacoplan, a small number of reports associated retinal vasculitis with drug administration (120, 121). This was investigated by the Research and Safety in Therapeutics Committee of the American Society of Retina Specialists, which could not identify a clear etiology for the vasculitis in these cases (122); overall, these cases have been very rare, and there is a very low risk of vasculitis with pegcetacoplan use (120).

A more compelling concern is the increased frequency of new-onset CNV in patients receiving either medication compared with sham treatment. In the GATHER1 trial, patients receiving 2 mg and 4 mg of avacincaptad had an 11.9% and 15.7% rate of new-onset CNV, respectively; their control groups exhibited a lower rate, at 2.7% and 2.4% (116). In OAKS and DERBY, there was a similar trend; in OAKS, after 24 months, CNV developed in 11% and 8% of eyes receiving pegcetacoplan monthly or every other month versus 2% of eyes in the sham treatment group (113). In DERBY, 13% and 8% of eyes receiving pegcetacoplan developed CNV versus 4% in the sham treatment group.

One hypothesis to explain this phenomenon is that in control groups, as the area of atrophy expands, the number of cells producing VEGF-A decreases, leading to lower intraocular VEGF-A levels and therefore less of a drive for CNV (123). In eyes receiving treatment, the rate of atrophy is decreased, preserving more cells, thereby maintaining a higher level of VEGF-A and promoting CNV (123). In a sense, the presence of new-onset CNV may be an indicator of the viability of the RPE and photoreceptor layer (123). In support of this hypothesis, one small observational study found a slower growth rate in GA lesion area in eyes with subclinical CNV compared with eyes without CNV (124).

An alternative hypothesis is that pharmacological C3 and C5 convertase inhibition leads to decreased levels of C3a and C5a, changing the intraocular signaling milieu and affecting polarization of resident macrophages such that there are more M2-like polarized proangiogenic macrophages and fewer proinflammatory M1-like polarized macrophages (35, 123, 125–127). In a study of human donor eyes, CNV lesions were indeed associated with the presence of activated macrophages, suggesting that macrophages could play a role in CNV formation (128). Interestingly, C3-deficient mice developed increased neovascularization in a model of retinopathy of prematurity; the same study found that macrophages stimulated with C5a displayed an antiangiogenesis phenotype, suggesting complement could play a role in regulating angiogenesis in the retina (129).

In the inverse of the above phenomenon (successful treatment of GA leading to CNV), long-term treatment of nvAMD with anti-VEGF therapy is sometimes associated with the development of GA (130), possibly secondary to choriocapillaris degeneration due to VEGF’s role in promoting endothelial cell survival or yet-unknown mechanisms, including progression of the underlying disease (62). VEGF may also have neurotrophic activity in the retina (131). This relationship among GA, CNV, VEGF, and complement inhibition will hopefully become clearer as more data emerge from ongoing trials of complement inhibition in the treatment of AMD.

### Investigational therapies in atrophic AMD.

Multiple drugs that target other complement components are in early clinical testing for AMD. Many of these drug trials are taking alternate approaches to treatment, such as gene therapy or oral administration. For example, JNJ-1887 (Janssen Pharmaceutical Co.; Table 3) is a gene therapy designed as a single intravitreal injection that increases expression of a soluble form of MAC-inhibitory protein (CD59) (Figure 1 and Table 3). JNJ-1887 is being studied in a phase II trial focused on patients with non-subfoveal GA; the primary end point is change from baseline GA lesion area (132).

ANX007 (Annexon Biosciences; Table 3) is a F(ab) fragment antibody that inhibits C1q, which binds antigen-antibody complexes and initiates the classical pathway of complement activation (Figure 1) (133). Interestingly, this drug was previously tested in glaucoma (ClinicalTrials.gov NCT0418815), but this indication appears to have been abandoned. ANX007 is being investigated in a phase II study in which it is administered every month or every other month via intravitreal injection to patients with GA (134). Results from the 12-month treatment period were announced in 2023, and while patients did not demonstrate a significant decrease in GA lesion area growth, they did demonstrate a significant reduction in risk of vision loss, suggesting a role of complement inhibition in neuroprotection (135). In support of this, animal models of retinal degeneration have shown that *C1qa^–/–^* mice have less photoreceptor cell death and improved electroretinogram responses compared with wild-type mice after exposure to photo-oxidative damage (136). Additionally, C1q has functions outside of complement activation; C1q signaling enhances phagocytosis and apoptotic cell clearance (137) as well as upregulating the antiinflammatory or M2 macrophage phenotype (138). These other roles of C1q could explain the seemingly contradictory result of C1q inhibition in dry AMD, i.e., lack of effect on GA growth but possible maintenance of photoreceptor integrity.

The blood-retina barrier prevents most systemic medications from reaching effective concentrations in the posterior segment of the eye, which is why the majority of potential therapeutics for GA are delivered intravitreally (139). Intravitreal administration, while effective, is invasive and carries risks such as endophthalmitis; therefore, drugs with alternate delivery routes are being pursued. ACH-4471 (Alexion Pharmaceuticals; Table 3) is a small-molecule FD inhibitor (140) that crosses the blood-retina barrier (141) and is being explored as an oral therapy for GA in a phase II trial (Figure 1) (142). Another potential oral therapy is iptacopan (FABHALTA, Novartis; Table 3), which inhibits FB, also being investigated in a phase II trial. IONIS-FB-L_RX_ (Ionis Pharmaceuticals; Table 3) is an antisense oligonucleotide that is administered subcutaneously and targets FB messenger RNA, reducing FB protein expression (143, 144).

AVD-104 (Aviceda Therapeutics) is a sialic acid–coated nanoparticle that targets both the humoral and cellular arms of the innate immune system (145). It binds FH directly and enhances the complement-inhibitory function of FH. It also binds to sialic acid–binding immunoglobulin-like lectin receptors on macrophages and triggers polarization to the M2, or an antiinflammatory/resolving phenotype. AVD-104 is currently being investigated in a phase II trial (Table 3).

The above drugs are examples of the innovative approach being taken to complement inhibition in the treatment of atrophic AMD. With so many therapies under investigation, conceivably there will be an array of options for treating atrophic AMD in the future.

## Conclusion

The complement system exists in a carefully balanced state within the eye; modulation of its activity is necessary for complement to perform its role as an innate immune effector while also maintaining a level of inflammation that does not interfere with retina structure and function. Complement dysregulation contributes to multiple ocular diseases and is a major contributor to AMD pathogenesis specifically. Investigation into complement activity in the posterior segment of the eye in the context of AMD has led to recent major advancements in therapies for atrophic AMD, a disease that was previously untreatable. This exploration of intraocular complement activity will hopefully continue to yield new insights into AMD and other vision-threatening diseases.

## Figures and Tables

**Table 2:**
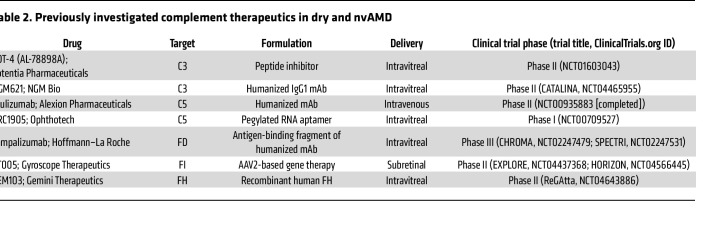
Previously investigated complement therapeutics in dry and nvAMD

**Table 1:**
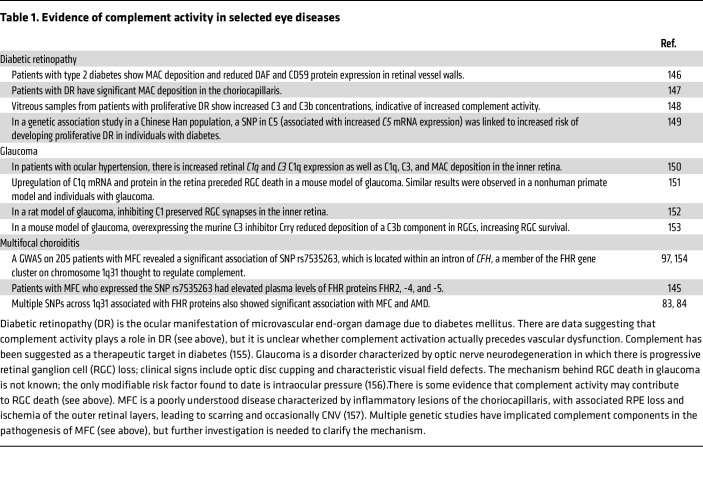
Evidence of complement activity in selected eye diseases

**Table 3:**
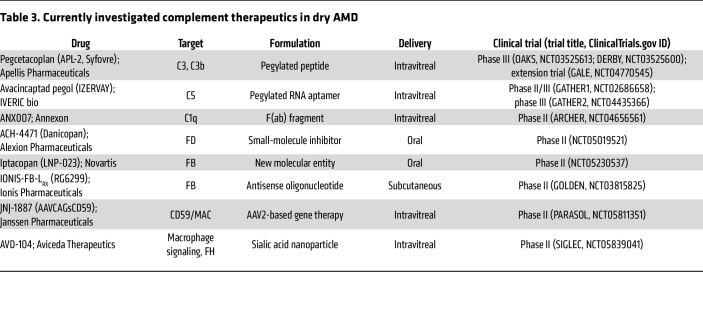
Currently investigated complement therapeutics in dry AMD

## Version 1 05/01/2024

Electronic publication
